# Development of trigger-based semi-automated surveillance of ventilator-associated pneumonia and central line-associated bloodstream infections in a Dutch intensive care

**DOI:** 10.1186/s13613-014-0040-x

**Published:** 2014-12-21

**Authors:** Anna Maria Kaiser, Evelien de Jong, Sabine FM Evelein-Brugman, Jan M Peppink, Christina MJE Vandenbroucke-Grauls, Armand RJ Girbes

**Affiliations:** 1 Department of Medical Microbiology and Infection Control, VU University Medical Centre, Amsterdam, 1007 MB, The Netherlands; 2 Department of Intensive Care, VU University Medical Centre, Amsterdam, 1007 MB, The Netherlands

**Keywords:** Hospital-acquired infection (HAI), Quality improvement, Electronic surveillance system, Decision support capabilities, Infection prevention (IP), Patient data management system

## Abstract

**Background:**

Availability of a patient data management system (PDMS) has created the opportunity to develop trigger-based electronic surveillance systems (ESSs). The aim was to evaluate a semi-automated trigger-based ESS for the detection of ventilator-associated pneumonia (VAP) and central line-associated blood stream infections (CLABSIs) in the intensive care.

**Methods:**

Prospective comparison of surveillance was based on a semi-automated ESS with and without trigger. Components of the VAP/CLABSI definition served as triggers. These included the use of VAP/CLABSI-related antibiotics, the presence of mechanical ventilation or an intravenous central line, and the presence of specific clinical symptoms. Triggers were automatically fired by the PDMS. Chest X-rays and microbiology culture results were checked only on patient days with a positive trigger signal from the ESS. In traditional screening, no triggers were used; therefore, chest X-rays and culture results had to be screened for all patient days of all included patients. Patients with pneumonia at admission were excluded.

**Results:**

A total of 553 patients were screened for VAP and CLABSI. The incidence of VAP was 3.3/1,000 ventilation days (13 VAP/3,927 mechanical ventilation days), and the incidence of CLABSI was 1.7/1,000 central line days (24 CLABSI/13.887 central line days). For VAP, the trigger-based screening had a sensitivity of 92.3%, a specificity of 100%, and a negative predictive value of 99.8% compared to traditional screening of all patients. For CLABSI, sensitivity was 91.3%, specificity 100%, and negative predictive value 99.6%.

**Conclusions:**

Pre-selection of patients to be checked for signs and symptoms of VAP and CLABSI by a computer-generated automated trigger system was time saving but slightly less accurate than conventional surveillance. However, this after-the-fact surveillance was mainly designed as a quality indicator over time rather than for precise determination of infection rates. Therefore, surveillance of VAP and CLABSI with a trigger-based ESS is feasible and effective.

## Background

Manual surveillance of hospital-acquired infections (HAIs) by infection prevention practitioners (IPPs) is very labor intensive and vulnerable to misclassification. According to Stone et al. [1], IPPs spend on average 45% of their working time on surveillance and analysis. Together with the trend towards mandatory and public reporting of HAI rates [2], this underscores the need for more effective surveillance efforts. Surveillance also secures a follow-up on the outcome of interventions directed at HAIs [3].

Over the last years, electronic surveillance systems (ESSs) for infection control programs have evolved rapidly and have led to a reduction of workload and thereby costs [1],[4] to improve efficiency, objectivity, and reproducibility of surveillance [5]-[7]. ESSs make surveillance more consistent and comparable [8]. This ongoing development of ESSs also benefits from developments of decision support capabilities [7],[9], classification algorithms [3],[10], and regression models [11].

The objective of this study was to evaluate an ESS for HAI surveillance with decision support capabilities in the intensive care unit (ICU), a so-called trigger-based ESS. We compared this screening method with our existing traditional daily screening of all admission days for signs and symptoms of HAI.

Since central line-associated bloodstream infection (CLABSI) and ventilator-associated pneumonia (VAP) are the most predominant HAI in an ICU setting [12], we chose these as outcome parameters. Also, VAP and CLABSI are notoriously difficult to diagnose which makes a consistent computer-based system potentially appealing [12],[13].

## Methods

### Setting

This study was conducted in the ICU department of VU University Medical Centre, Amsterdam. The ICU is a mixed ICU with 24 beds. In nearly all hospitals in the Netherlands, selective decontamination of the digestive tract (SDD) is used as standard prophylaxis for VAP in patients admitted for >48 h.

In our hospital, SDD consists of cefotaxime iv during the first 3 days of stay in the ICU and enteral administration of 64 mg tobramycin, 100 mg colistin, and 500 mg amphotericin B four times daily. A paste containing tobramycin 2%, amphotericin B 2%, and colistin 2% is applied to the buccal mucosa four times daily.

### Ethical approval

Surveillance of nosocomial infections is part of the regular quality control program of the hospital, performed in compliance with the Helsinki Declaration and as such waved by the institutional review board.

### Data collection

Patient data are stored in a hospital management database (HMDB) system, a clinical data warehouse. Patient data include age, sex, date of admission and discharge, radiology reports, blood parameters, and microbiological results. In addition, the ICU has a dedicated patient data management system (PDMS; MetaVision®, iMDsoft, Tel Aviv, Israel). The PDMS contains all relevant information related to the ICU and integrates automatically relevant patient-related data derived from the HMDB. Since 2005, the PDMS and HMDB are used for VAP surveillance in the ICU. Data collection in the PDMS is mostly automated (e.g., blood pressure, ventilation data) by a direct link to the equipment. Orders and specifications as given by doctors and nurses (e.g., orders for antibiotics, description of sputum appearance) are entered into the PDMS manually. Data collection in the PDMS is according to ICU quality protocols and standards. The information technology expert on our ICU has developed a program (VB.net), which runs weekly. VB.net retrieves the daily surveillance-relevant data from the PDMS and transforms them into scores. Then, the algorithm determines if there is a VAP/CLABSI trigger per day, and finally, the data are added to the Access database.

### Surveillance methods

**Figure 1:**
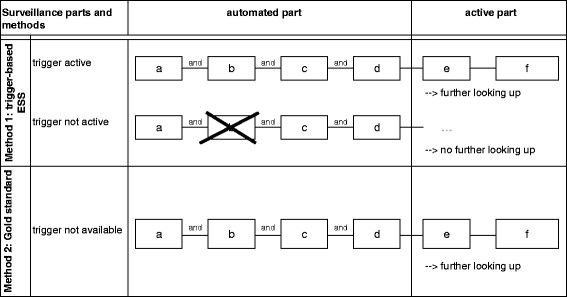
**Illustration of how automated and active parts trigger surveillance.** Legend: a-d, factors relevant for trigger activation; e,f, factors of the active part of the surveillance.

From October 2009 till October 2010, surveillance of VAP and CLABSI was conducted both with trigger activation (trigger-based ESS) and without trigger activation (gold standard). An infection prevention research coordinator performed the surveillance, and the presence of VAP or CLABSI was assessed in cooperation with an experienced intensive care physician. In the trigger-based ESS, patients with a possible VAP or CLABSI were pre-selected by a trigger signal (automated part), which showed up when all criteria of the automated part were fulfilled (based on factors a to d, see Figure 1). These automated factors were clearly defined in the PDMS and needed no manual interpretation. The algorithm determined daily whether to fire a trigger for VAP or CLABSI. Data that had activated the trigger were retrieved automatically into the Access database. The remaining criteria (factors e and f, see Figure 1) to be met to confirm the presence of VAP or CLABSI were then checked manually, only for the patients with an active trigger. For traditional screening without trigger, factors e and f were checked manually for *all* patients under surveillance every day. Data on factors e and f were entered in the Access database by the research coordinator. The manual screening for criteria e and f was performed approximately 2 weeks after discharge of the patient, when results of microbiological cultures were known and radiology reports were accessible. The active part of the surveillance (checking of microbiological results and/or X-rays results) required approximately 1 min per patient per day and per VAP/CLABSI. In traditional screening, we totaled all patient days to calculate the workload. In the trigger-based ESS, only triggered patient days were taken into account. All patient days where the trigger did not fire represent saved time.

### Definitions of VAP/CLABSI

**Figure 2:**
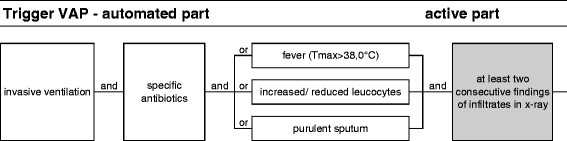
Diagnostic algorithm to identify cases of VAP.

**Figure 3:**
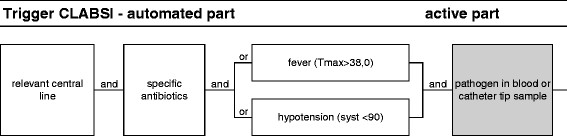
Diagnostic algorithm to identify cases of CLABSI.

CDC definitions [14] of VAP and CLABSI were slightly amended to allow automatic assessment (see Figures 2 and 3). Only the first episode of CLABSI or VAP per admission was included, as it is difficult to distinguish the beginning and end of a separate episode.

For VAP, the trigger was activated when the patient had mechanical ventilation and was given specific antibiotics for VAP treatment (ceftriaxone, levofloxacin, ciprofloxacin, sulfamethoxazole and trimethoprim (cotrimoxazole), ceftazidime, tobramycin, gentamicin, imipenem, fluconazole, and voriconazole), in combination with one of the following clinical symptoms: temperature above 38°C, white blood cell count <4,000 or >12,000/mm^3^, and presence of purulent sputum. For every occurring sputum, nurses fill in the PDMS with descriptions such as optionally purulent, white, or yellow sputum. The CDC sign ‘Worsening of gas exchange’ was not used. The active part included the presence of lung infiltrates/consolidations in radiology reports on at least two consecutive days. Rapidly disappearing (<1 day) densities on the chest X-ray were considered not to be related to VAP but to (hydrostatic) transient edema. These X-ray findings were verified by the radiologist. The definitive diagnosis was confirmed in cooperation with the experienced intensive care physician. When the diagnosis of VAP was not conclusive, the results of sputum/BAL cultures and respiratory deterioration were taken into account.

To indicate suspected CLABSI, the trigger was activated when one of the following central lines was present - arterial, dialysis, single lumen, double lumen, triple lumen, and quadruple lumen - and when CLABSI-specific antibiotics were used (vancomycin, flucloxacillin, ceftriaxone, ciprofloxacin, imipenem, fluconazole, and voriconazole), in combination with one of the following clinical symptoms: temperature above 38°C and hypotension with a systolic pressure <90 mmHg (see Figure 3). The CDC sign ‘presence of cold shivers’ for CLABSI was not used. The active part included the evaluation of the culture results of blood and central line samples. The microbiological culture policy for CLABSI was as follows: in case of temperature above 38°C or increase in the abovementioned clinical symptoms, blood samples were taken from each intravenous central line that was in place at that moment. If one or more central lines were removed, the tip was sent for culture. At least ten colonies of the pathogen had to be found optionally in a catheter tip sample. At least one catheter tip or blood sample had been taken from all patients with clinical symptoms for CLABSI. Catheter tip colonization was diagnosed by a qualitative method: the catheter tip was vigorously shaken in 1 ml sodium chloride, and 100 μl of this suspension was subcultured on agar plates. Growth was reported as +, ++, or +++. Culture results were interpreted in the context of the clinical signs and symptoms (contaminant vs pathogen) and the presence of other foci of infection.

### Patient selection

We included patients with a minimal length of ICU stay (LOS) of 48 h and evaluated for a maximum of 8 weeks of stay. Patients with pneumonia on admission were excluded. The presence of mechanical ventilation, the use of specific antibiotics, and clinical criteria were retrieved daily from the PDMS. Central line days and ventilation days, respectively, were registered automatically until ICU discharge or death; in case of CLABSI or VAP, they were registered until the day of infection. Patients readmitted to the ICU >24 h after discharge were considered new patients. Patients in whom life-prolonging treatment was stopped were excluded from the analysis from that moment on, taking into account that no further diagnostics is done nor specific antibiotic treatment is given anymore.

### Missing values

Factors of the automated part of the surveillance were mandatory: no missing data were allowed. In cases that there was no information on the presence of pneumonia on admission, this field was corrected during the active phase of the surveillance (based on the patient's chart). Sometimes, administrative data (in particular for history of the patient) were missing. This, however, would not affect the results [15].

### Statistical analysis

Analysis was performed with SPSS® version 15 (SPSS Inc., Chicago, IL, USA) and Microsoft Excel® (Microsoft Office 2010, Microsoft Corporation, Redmond, WA, USA). The two screening methods were compared by calculating the sensitivity, specificity, and positive and negative predictive values.

## Results

During the study period, 553 patients were included, with a total of 6,793 patient days of stay (see Table 1). Two patients were excluded from the study for further analysis from the moment that life-prolonging treatment was stopped, accounting for 28 patient days (0.4%). In our setting, all patients identified with VAP or CLABSI through the trigger screening were also identified through traditional screening (positive predictive value: 100%); all patients with no VAP or CLABSI in trigger screening were also identified as such through traditional screening (specificity: 100%), as a reference gold standard.

**Table 1:** **Patient characteristics (*n*** **= 553)**

	**Value**
Age (year), mean, SD, range	62.5 ± 15.8 (16 to 96)
Gender (male), mean, range	343 (62%) (16 to 96)
APACHE II score, mean, SD, range	24.2 ± 7.9 (0 to 49), 46 missing
ICU length of stay (days), mean, SD, range	11.7 ± 11.6 (2 to 56)
ICU mortality, *n* (%)	127 (23%)
Pneumonia on admission, *n* (%)	131 (25%)

Mean (SD), or number (percentage), when appropriate.

### VAP

A total of 131 patients were diagnosed with pneumonia on admission to the ICU and were excluded from the VAP registration. Thirteen of the remaining 422 patients (3.0%) acquired VAP during their stay. The patients had a total length of stay of 6,793 days; on 359 (5.3%) of these days, the ESS showed a VAP trigger. With 3,927 mechanical ventilation days, the incidence of VAP in traditional screening was 3.3/1,000 ventilation days, (95% confidence interval (CI): 1.5 to 5.1). By traditional screening, VAP was diagnosed in 13 patients (see Table 2). One of these patients was not identified by the trigger system and hence was not diagnosed with VAP. For this patient, the treatment was started 3 days after the development of clinical symptoms and infiltrates on X-ray, so the trigger went off too late. The VAP incidence determined with the trigger system was 3.1/1,000 ventilation days, (95% CI: 1.3 to 4.8). The sensitivity of the trigger screening was 92.3% (95% CI: 63.9% to 98.7%). The trigger-based ESS correctly identified 409 out of the 410 patients who did not develop VAP. This finding results in a negative predictive value of 99.8% (95% CI: 98.6% to 100%) for the VAP trigger. All patients with VAP in trigger screening were confirmed by traditional screening (100% positive predictive value), and all patients without VAP with trigger screening had no VAP according to traditional screening (100% specificity). In 359 of the 6,793 patient days (5.3%), the VAP trigger was activated.

**Table 2:** The performance of a trigger screening for detection of VAP and CLABSI

	**Group 2 (gold standard)**			**Sensitivity, % (95% CI)**	**Specificity, % (95% CI)**	**PPV, % (95% CI)**	**NPV, % (95% CI)**
Group 1 (trigger-based)	VAP	No VAP	Total VAP	12/13	409/409	12/12	409/410
VAP	12	0	12	92.3%	100.0%	100.0%	99.8%
No VAP	1	409	410	(63.90 to 98.72)	(99.09 to 100.00)	(73.35 to 100.00)	(98.64 to 99.96)
Total VAP	13	409	422				
	CLABSI	No CLABSI	Total CLABSI	22/24	529/529	22/22	529/531
CLABSI	22	0	22	91.7%	100.0%	100.0%	99.6%
No CLABSI	2	529	531	(72.96 to 98.73)	(99.30 to 100.00)	(84.43 to 100.00)	(98.64 to 99.94)
Total CLABSI	24	529	553				

PPV, positive predictive value; NPV, negative predictive value.

### CLABSI

Of the 553 inclusions, 24 patients (4.3%) acquired a CLABSI during admission in the ICU during 13.887 central line days. The incidence in traditional screening for CLABSI was 1.7/1,000 central line days (95% CI: 1.0 to 2.4). Of the 6,793 patient days, 983 days were with an active CLABSI trigger (14.5%). By traditional screening, CLABSI was diagnosed in 24 patients. Two of them were not identified by the trigger system. Their central line had been removed, and they had not received antibiotic treatment.

The CLABSI incidence with the trigger system was 1.6/1,000 central line days, (95% CI: 0.9 to 2.2). The sensitivity of the trigger screening was 91.3% (95% CI: 73.0% to 98.7%), as 22 out of 24 CLABSI were identified through trigger screening (see Table 2). Five hundred twenty-nine of the total 531 inclusions without CLABSI were identified by the trigger system. This finding results in a negative predictive value of 99.6% (95% CI: 98.6% to 99.9%) for the CLABSI trigger screening. All patients with CLABSI in trigger screening were confirmed by traditional screening (100% positive predictive value), and all patients without CLABSI with trigger screening had no CLABSI according to traditional screening (100% specificity).

In 983 of the 6,793 patient days (14.5%), the CLABSI trigger was activated. Together for VAP and CLABSI, trigger activation occurred in about 20% of the patient days, both for VAP (5.3% active trigger) and CLABSI (14.5% active trigger).

Total manual labor time for traditional screening amounted to 226 days (time needed for checking of 6,793 patient days of stay in the ICU). This makes 4.4 h/week for checking for both CLABSI and VAP. Trigger-based screening reduced labor time to 22 h for 1,342 days, since triggers were activated on 1,342 of the 6,793 patient days, 359 times for VAP and 983 times for CLABSI. For VAP, labor time with trigger screening was reduced from 2.2 to 0.3 h/week, and for CLABSI, labor time with trigger screening was reduced from 2.2 to 0.1 h/week. In summary, manual labor time for screening and determination of infection was reduced by 90% from an original 4.4 h per week to 26 min per week.

## Discussion

In this study, we compared detection of VAP and CLABSI on the ICU by two surveillance methods: one with a trigger-based ESS and one without a trigger-based ESS (gold standard). The trigger-based ESS showed high sensitivity and negative predictive value when compared with the system without trigger.

Compared to the gold standard, the trigger-based ESS resulted in a workload reduction of 90%. This is a rough estimation of saved time. The principle of a trigger-based surveillance is not new: Klompas et al. developed in 2008 an algorithm for surveillance of VAP, where only patients who met ventilator-change criteria were examined further to see whether they fulfilled the remaining criteria for VAP [8]. In 2011, Klompas used ventilator-associated complications (VACs) as a faster and more objective predictor of outcomes versus VAP [13]. Woeltje et al. developed an automated CLABSI surveillance based on laboratory data where combinations of dichotomous prediction rules were applied to electronic data [10]. Because not all types of CLABSI require positive microbiology results to meet case definitions, basing the initial case finding on microbiology results only will lead to lower sensitivity of detection. This is also dependent on the performance of diagnostic testing [4]. In the system that we developed for CLABSI surveillance, microbiology results were not used as components of the trigger system but were used in the active part of the surveillance. This was done to interpret the microbiological results in the context of the diagnoses of CLABSI. A trigger system will not be suitable for every type of infection or every department in a hospital. The higher the incidence of an outcome parameter, the more frequently the trigger will be activated. Hence, an automated trigger surveillance system is most time effective in case of a low incidence of the outcome parameter. In our ICU, the incidence of VAP is very low, related to the use of SDD and strict adherence to guidelines on oral hygiene and bronchial suction. In several studies, SDD has shown to be very effective in reducing the incidence of VAP [16]. This trigger-based ESS is developed for the purpose of retrospective HAI surveillance, not for real-time surveillance. The trigger pre-selects potential cases of VAP and CLABSI based on a simple algorithm of clinical treatment factors. VAP and CLABSI definitions however are difficult to apply objectively [12]. To make surveillance more consistent and reproducible, we used slightly modified VAP/CLABSI criteria. This might have influenced the outcome: surveillance HAI definitions are commonly less specific than the clinical diagnosis [8],[12].

One of the limitations of the trigger-activated ESS that we propose is that the trigger activation depends on the strict adherence of ICU physicians to the antibiotic policy that is agreed for treatment of VAP and CLABSI. A second drawback is that infections that are not treated with antibiotics will go undetected. This probably is not really an issue for VAP but might lead to underestimation of CLABSI, because a possible CLABSI might be treated by simple central line removal without antibiotic treatment. In our ICU, rigorous guidelines are used for antibiotic treatment of VAP and CLABSI. Therefore, we were able to base the algorithm that fires the trigger on the start of antibiotic treatment. In addition, clinical parameters that are important in the diagnosis of VAP and CLABSI are recorded routinely in the PDMS. This permitted the inclusion of these parameters in the automated and hence objective part of the algorithm. The manual part included the evaluation of radiographic signs for VAP and the interpretation of blood culture results for CLABSI. This interpretation can be subjective, and therefore, for all VAP and CLABSI, it was performed by two persons and discussed until consensus was reached. As our study is a surveillance after the fact, it has no influence on an individual patients' treatment, and therefore, the missing two cases of CLABSI would not appear as a significant problem.

Another weakness of our surveillance is the short study period and the low number of VAP and CLABSI cases, and the high sensitivity and negative predicted values we measured might be due to this limitation [17].

Future steps will be to create a uniform comparable surveillance system, taking into account that the trigger will have to be adjusted for every hospital according to the local antibiotic policy [11].

Further reduction of the manual part of the surveillance system remains the next goal. Haas et al. [18] evaluated the utility of natural language processing to search radiographic reports for descriptions suggestive of pneumonia in a neonatal intensive care unit. Another challenge is real-time surveillance [4]. This is hampered by the lack of real-time availability of complete microbiological culture results and radiographic reports.

Van Mourik et al. [11] showed that models with multiple indicators or predictors of drain-related meningitis simultaneously achieved near-perfect sensitivity. However, HAI definitions require a clinical judgement [12], and automated data alone are not sufficient to confirm their presence [19]. Mayer et al. [20] showed that agreement in classifying CLABSI among multiple reviewers conducting surveillance is poor. She emphasized that the reliability of a non-fully automated surveillance to identify hospital infections may be ideal for surveillance within a hospital, but not for inter-hospital comparisons.

## Conclusions

Pre-selection of patients to be checked for signs and symptoms of VAP and CLABSI by a computer-generated automated trigger system was time saving but slightly less accurate than conventional surveillance. However, this after-the-fact surveillance was mainly designed as a quality indicator over time rather than for precise determination of infection rates. Therefore, surveillance of VAP and CLABSI with a trigger-based ESS is feasible and effective.

## Competing interests

The authors declare that they have no competing interests.

## Authors’ contributions

AK coordinated the study, drafted the manuscript, collected the data, and performed the statistical analysis. JP designed the database. AK, SE, CG, and AG participated in the design of the database. EJ, CG, and AG helped in the drafting of the manuscript for important intellectual content. All authors read and approved the final manuscript.

## Authors’ information

AK has a MSc degree. CG and AG have PhD and MD degrees. EJ and SE have a MD degree.
